# Clinical phenotypic and genotypic characterization of *NPRL3*-related epilepsy

**DOI:** 10.3389/fneur.2023.1113747

**Published:** 2023-03-02

**Authors:** Hongwei Zhang, Jie Deng, Xiaohui Wang, Chunhong Chen, Shuhua Chen, Lifang Dai, Fang Fang

**Affiliations:** ^1^Department of Neurology, Beijing Children's Hospital, Capital Medical University, National Center for Children's Health, Beijing, China; ^2^Epilepsy Center, Children's Hospital Affiliated to Shandong University, Jinan, China; ^3^Epilepsy Center, Jinan Children's Hospital, Jinan, China

**Keywords:** *NPRL3*, epilepsy, genotype, phenotype, treatment

## Abstract

**Background:**

As one of the assembly factors of the GATOR1 protein complex in the mechanism of rapamycin pathway, NPRL3 plays an important role in the pathogenesis of epilepsy. However, the correlation between genotype and clinical phenotype in patients with *NPRL3*-related epilepsy has not been clarified.

**Methods:**

A total of 11 Chinese children with *NPRL3*-related epilepsy were identified through whole-exome sequencing (WES). The data from the clinical presentation, laboratory data, brain imaging findings, genetic results, and treatment methods were collected. All previously reported cases with *NPRL3*-related epilepsy were collected and reviewed through PubMed search.

**Results:**

Among the 11 children, eight have not been reported, and two of them presented infantile spasms (ISs) as a new phenotype of *NPRL3*-related epilepsy. In addition, WES identified five frameshift mutations, three nonsense mutations, two missense mutations, and one exon deletion. Based on bioinformatics analysis, it was found that two missense mutation sites were highly conserved, and the c.400G>A mutation site of the *NPRL3* gene caused the alteration of the protein structure. To date, 88 patients have been reported with *NPRL3*-related defects, including our 11 cases. The most common presentations were sleep-related hypermotor epilepsy (SHE), frontal lobe epilepsy (FLE), and temporal lobe epilepsy. A majority of patients (70%) presented normal neuroimaging results, and focal cortical dysplasia was the most common neuroimaging abnormality (62.5%). Among the *NPRL3* gene mutations, loss of function (nonsense mutations, frameshift mutations, and exons deletion) was the most common genetic variation (75%). For 73% of patients with *NPRL3*-related epilepsy, monotherapy of sodium channel blockers was effective. Surgery was effective for 75% of children with neuroimaging abnormalities. Two cases unresponsive to surgery or anti-seizure medications were treated with ketogenic diets (KD), which were effective. One case was treated with rapamycin at an early stage of epilepsy, which was effective as well.

**Conclusion:**

*NPRL3*-related epilepsy has high clinical and genetic heterogeneity. SHE and FLE are the most common clinical presentations. Furthermore, ISs are the new phenotypes of *NPRL3*-related epilepsy, while the variants c.275G>A, c.745G>A, and c.1270C>T may be the most common *NPRL3* gene mutations. Sodium channel blockers, surgery, KD, and rapamycin may be the potential treatments for these patients. Our study expanded the clinical and genetic spectrum of *NPRL3*-related epilepsy and provided important information for the precise treatment of patients.

## Introduction

Epilepsy is a common childhood neurological disorder that occurs in more than 50 million people worldwide (1), and 5 million new cases are reported each year (2). It is characterized by recurrent, unprovoked seizures due to abnormal synchronized neuronal firing in the brain (3). The etiology of epilepsy is complex and varied, with genetic factors accounting for 40% (4). More than 500 genes involved in epilepsy have been reported (5), indicating the genetic heterogeneity of epilepsy. In the etiology of epilepsy, novel gene discoveries have moved the field beyond the known contribution of ion channels to implicate chromatin remodeling, transcriptional regulation, and regulation of the mammalian target of rapamycin (mTOR) protein (3). Indeed, such discoveries pave the way for finding new therapeutic targets, some of which have already been studied (3). Thus, it is of great value to explore the correlation between genotype and clinical phenotype of an mTOR signaling pathway in the field of pediatric epilepsy.

Nitrogen permease regulator-like 3 (NPRL3), a component of the GATOR1 complex that negatively regulates the mTOR signaling pathway, reportedly associates with brain development and function (6). The *NPRL3* gene, which is located on chromosome 6p13.3 connecting to the *NPRL2* gene and encodes a protein containing 569 amino acids, forms the GATOR1 complex together with DEPDC5 and nitrogen permease regulator like-2 (NPRL2) (6). Its transcript is highly expressed in the brain's frontal, temporal, parietal, and occipital lobes, similar to the distribution pattern to that of DEPDC5 and NPRL3 transcriptions (3, 7). Pathogenic variants in the *NPRL3* gene can cause loss of function of the GATOR1 complex, thereby abnormally enhancing the activity of the mTOR signaling pathway (8). Recently, an increasing number of presumably pathogenic mutations of the *NPRL3* gene were reported in the cases of focal epilepsy (7, 9, 10), such as frontal lobe epilepsy (FLE) (11), benign epilepsy in children with centrotemporal spikes (BECT), and familial focal epilepsy with variable foci (FFEVF) (12, 13). These manifestations resemble clinical phenotypes observed in patients with pathogenic variants in the DEPDC5 and *NRPL2* genes (8). However, the details on whether the new phenotypes and genotypes of the *NPRL3* gene do exist and the information on the correlation between genotype and clinical phenotype in patients with *NPRL3*-related epilepsy have not been clarified. In addition, previous studies showed that more than half of the patients with epilepsy due to GATOR1 variants had drug resistance to traditional anti-seizure medications (ASMs) (14). The current treatment of epilepsy with *NPRL3* gene variants is lacking in specificity. Therefore, we described the clinical characteristics of 11 patients with different novel-likely pathogenic variants in the *NPRL3* gene, then reviewed the advanced publications, and finally summarized the associations between phenotype and genotype in *NPRL3*-related epilepsy to provide a reference for the precise treatment of patients with *NPRL3*-related epilepsy.

## Materials and methods

### Patients

In this study, 11 children with *NPRL3*-related epilepsy were recruited from the Department of Neurology at Beijing Children's Hospital, Capital Medical University and Epilepsy Center at Jinan Children's Hospital from March 2018 to May 2022. The clinical presentation, laboratory data, brain imaging findings, and genetic results were collected and reviewed through electronic medical records and telephone follow-ups. All procedures followed in this study were approved by the institutional ethics committee of Beijing Children's Hospital, Capital Medical University (2019-k-262). All blood samples were collected after obtaining written consent from the parents of each patient in compliance with the Declaration of Helsinki.

### Whole-exome sequencing and Sanger validation

Genomic DNA was extracted using a Relax Gene Blood DNA system (Tiangen Biotech Co., Ltd., Beijing China). The libraries for WES were constructed with Nano Prep DNA Library Preparation Module (for MGI), 96 rxn, and then sequenced on a BGI MGISEQ-2000 sequencer in 2 × 150 bp paired-end reads at a minimum of 150 × coverage. After sequencing, the read alignment was performed using the Burrows–Wheeler Aligner tool, version 0.7.17, with default parameters against the human genome assembly hg19 (GRCh37). The generated bam file was sorted and deduplicated by SAMtools and Picard, respectively. Then, Genome Analysis Toolkit was applied to detect SNVs and indels (< 50 bp), and CNVkit was performed to detect the copy number variations (CNVs). The 1000 Genome Project, Genome Aggregation Database, Exome Aggregation Consortium, and others were employed to annotate the variants in the frequency of the population. Furthermore, the Online Mendelian Inheritance in Man (OMIM) and the Human Gene Mutation Database (HGMD) were used to annotate the related diseases. With regard to the possible effects on protein function, the variants were evaluated by four widely used prediction tools, namely, Polymorphism Phenotyping version 2 (PolyPhen-2), Sorting Intolerant from Tolerant (SIFT), MutationTaster, and Splice AI. The pathogenicity of variation loci was analyzed according to the genetic variation classification criteria and guidelines proposed by the American College of Medical Genetics and Genomics (ACMG) (15). Based on the new guidelines for clinical interpretation of GATOR1 variants (14), missense variants with the Genome Aggregation Database (gnomAD) frequency within the pathogenic range threshold and predicted possibly pathogenic by M-CAP were defined as likely pathogenic. Sanger sequencing was performed to validate the variations and determine their parental origin, and the primer information was shown in Table 1.

**Table 1:** The primer sequences of different mutation sites of *NPRL3* gene.

**Primer name**	**Forward primer sequence**	**Reverse primer sequence**
c.1195C>T (p.Gln399Ter)	GGACACGAGGCACGTGCAT	CTCTCCCTTCCCAGGAGGGTG
c.763delA (p.Ile255SerfsTer28)	GGATGCGGGACAACACCA	GAGCAGATCTCGAATGCCCA
c.511delC (p.Leu171TrpfsTer46)	CTGAATGACAGCCCCTCCAC	CAGGCATTTCCAGAGCCATC
c.81delC (p.Phe28SerfsTer59)	TCCGCAGTGGGAAGAAGAAA	AGCACCATTGAGCAGGGTGT

### Bioinformatics analysis

In this study, we assessed the conservation of two variant sites by the University of California Santa Cruz (UCSC) online database (https://genome.ucsc.edu/) and constructed a tertiary structure model of the NPRL3 to analyze whether gene mutations cause the protein structure alteration. Specifically, the sequence of NPRL3 was available from the online protein database (https://www.rcsb.org); therefore, we selected the model with the highest C-score for download on Iterative Threading ASSEmbly Refinement (I-TASSER). Then, we selected a suitable template from a known protein structure to match the amino acid residues of NPRL3 on the template by sequence alignment. The structure of the wild-type NPRL3 was obtained from the AlphaFold Protein Structure Database (https://www.alphafold.ebi.ac.uk/), and the tertiary structure of the mutant NPRL3 was obtained by the PyMOL2.3 software.

### MR techniques and protocols

Brain MRI of both patients was conducted either at the Department of Neurology of Beijing Children's Hospital, Capital Medical University or at the Epilepsy Center at Jinan Children's Hospital and included high-resolution spin-echo T1-weighted images, spin–echo T2-weighted images, T2-weighted images with suppression of the cerebrospinal fluid (CSF) signal [fluid-attenuated inversion recovery (FLAIR)], and diffusion-weighted images sensitive to water diffusivity.

### Literature review

A systematic literature search of previously reported cases of *NPRL3*-related epilepsy was carried out in the PubMed database. MeSH and title or abstract were used for all eligible studies that mainly focus on the *NPRL3* variants in epilepsy. The research strategy was as follows: “NPRL3” AND (“epilepsy” or “infantile spasms”). The clinical presentation, laboratory data, brain imaging findings, genetic results, and treatment methods were obtained from the respective references, reviewed, and compared with those of our study.

## Results

### Clinical phenotype

**Figure 1:**
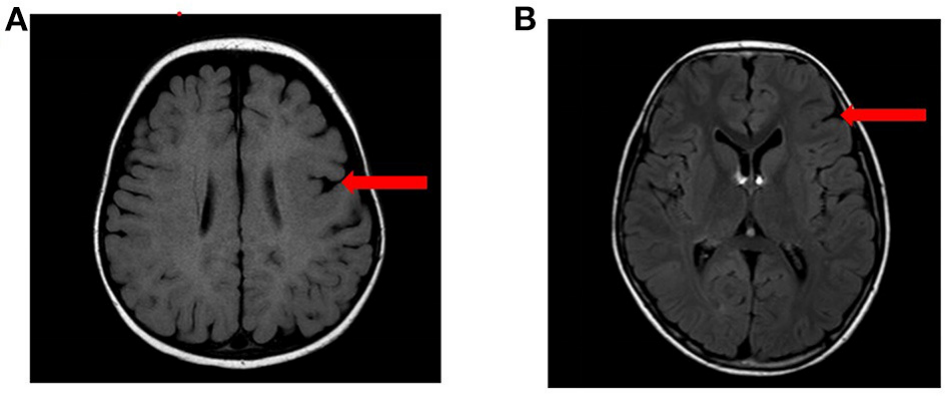
The MRI results of the patients. **(A)** MRI results of case 3: left frontal MCD. **(B)** MRI results of case 10: left frontal FCD.

Among the 11 patients with epilepsy, 9 cases were boys and 2 were girls. The age of epilepsy onset ranged from 4 months to 6 years and 10 months. Among the 11 patients, 5 cases were diagnosed with sleep-related hypermotor epilepsy (SHE) (cases 1–3, 6, and 11), 2 cases were FLE (cases 9 and 10), 2 cases were ISs (cases 4 and 8), and 2 cases were unclassified (cases 5 and 7). Seizure duration ranged from 3 s to 20 min, and seizure frequency ranged from one time every 1–2 months to more than 10 times per day. The craniocerebral MRI revealed that case 3 had left frontal malformation of cortical development (MCD), and case 10 had left frontal focal cortical dysplasia (FCD) (Figure 1). The cognitive assessment revealed that four patients had normal cognitive function (cases 2, 5, 9, and 10), two patients had roughly normal cognition (cases 7 and 11), two patients had mild cognitive impairment (cases 1 and 6), two patients had severe cognitive impairment (case 3 and 8), and one patient had an extreme developmental delay (case 4). Three patients had slightly low muscle tone (cases 2, 3, and 8). Two patients showed psycho-behavioral abnormalities with attention deficit (cases 6 and 9). Two patients had a family history of epilepsy (cases 1 and 9), and all patients had no sudden unexpected death in epilepsy (SUDEP) in their families. Five patients had drug-refractory epilepsy (cases 3, 4, 8, 10, and 11). Six patients had seizures controlled by effective treatments, including five patients with sodium channel blockers such as oxcarbazepine (OXC), lamotrigine (LTG), and lacosamide (LCM) (cases 1, 2, 6, 7, and 9), and one patient with ketogenic diet (KD) therapy (case 3) as shown in Table 2.

**Table 2:** The genotypes and clinical phenotypes of 11 children with *NPRL3*-related epilepsy.

**Case**	**Gender **	**cDNA/protein alteration**	**Variant class **	**ACMG class **	**Segregation analysis**	**Age at onset**	**Seizure frequency **	**Epilepsy syndrome**	**MRI **	**EEG discharge region**	**Cognitive function**	**ASMs or KD **	**Effective treatment **	**Epilepsy surgery**	**Seizure-free**	**SUDEP **	**Drug-resistant**
1	M	c.78C>A/p.Y26X	Nonsense	P	Inherited	1 y 4 m	More than 10 times daily	SHE	No	Central and frontal region	Mildly delayed	LEV, NZP, LCM, PB, VPA, OXC	LCM	No	Yes	No	No
2	M	c.1195C>T/p.Gln399Ter	Nonsense	LP	Inherited	1 y 10 m	Several times daily	SHE	No	Bilateral frontal and central region	Normal	OXC, CZP, LEV, VPA	LEV + VPA	No	Yes	No	No
3	F	c.274C>T/p.R92X	Nonsense	P	Inherited	4 m	Several times daily	SHE	Left frontal MCD	Bilateral multi-focal region	Severely developmentally delayed	LEV, OXC, TPM, NZP, LCM, VPA, PB, KD	KD	Yes	No	No	Yes
4	M	c.400G>A/p.Ala134Thr	Missense	VUS	Inherited	6 m	Several times daily	IS	No	Bilateral posterior region	Extreme developmentally delayed	CLB, CZP, TPM, VGB, VPA, ACTH, LEV, LTG, KD	LTG	No	No	No	Yes
5	M	c.649G>A/p.V217I	Missense	VUS	Inherited	2 y 3 m	Once every 20 days	Unclassified	No	Anterior and left central region	Normal	VPA, OXC	N/A	No	No	No	N/A
6	M	c.763delA/p.Ile255SerfsTer28	Frameshift	LP	Inherited	2 y 1 m	7–8 times daily	SHE	No	Bilateral frontal and temporal region	Mildly delayed	OXC, VPA, PB, TPM, LTG, VPA	LTG	No	Yes	No	No
7	F	c.1226-1227delAT/p.Y409CfsTer46	Frameshift	P	De novo	7 m	2–9 times daily	Unclassified	No	Bilateral occipital and temporal region	Roughly normal	OXC	OXC	No	Yes	No	No
8	M	c.1128del/p.E376DfsTer37	Frameshift	LP	Inherited	9 m	Several times daily	IS	No	Bilateral posterior region	Severely delayed	ACTH, VGB, VPA, CZP	No	No	No	No	Yes
9	M	c.511delC/p.Leu171TrpfsTer46	Frameshift	LP	Inherited	6 y 10 m	2–5 times daily	FLE	No	Bilateral frontal and temporal region	Normal	TPM, VPA, NZP, OXC, LCM	LCM	No	Yes	No	No
10	M	c.81delC/p.Phe28SerfsTer59	Frameshift	LP	Inherited	4 y	Once every 1–2 months	FLE	Left frontal FCD	Left frontal region	Normal	LCM, CZP, LEV, LTG, OXC	No	No	No	No	Yes
11	M	Deletion (exon 5-6)/p. (amino acid)	Exon deletion	LP	Inherited	1 y	2–5 times daily	SHE	No	Bilateral posterior region	Roughly normal	VPA, LTG, TPM, LEV, NZP	No	No	No	No	Yes

ACTH, adrenocorticotropic hormone; ASMs, antiseizure medications; CBZ, carbamazepine; CLB, clobazam; CZP, clonazepam; F, female; FCD, focal cortical dysplasia; FE, focal epilepsy; FLE, frontal lobe epilepsy; IS, infantile spasms; KD, ketogenic diet; LCM, lacosamide; LP, likely pathogenic; LTG, lamotrigine; M, male; MCD, malformations of cortical development; N/A, not applicable; NZP, nitrazepam; OXC, oxcarbazepine; P, pathogenic; PB, phenobarbital; SHE, sleep-related hypermotor epilepsy; SUDEP, sudden unexpected death in epilepsy; TPM, topiramate; VUS, variant of uncertain significance; VGB, vigabatrin; VPA, valproic acid.

### *NPRL3* gene pathogenic variant detection and gene analysis

In the 11 patients with epilepsy, all *NPRL3* gene variants were heterozygous. Five of the *NPRL3* gene variants were frameshift mutation (cases 6–10), three were nonsense pathogenic variants (cases 1–3), two were missense pathogenic variants (cases 4 and 5), and one was deletion (case 11). Three cases carrying pathogenic variants were internationally reported variations (case 1, p.Y26X; case 3, p.R92X; and case 7, p.Y409Cfs^*^46). The remaining eight new variation sites were identified and first reported, including p.Gln399Ter, p.Ala134Thr, p.Val217Ile, p.Ile255SerfsTer28, p.E376Dfs^*^37, p.Leu171TrpfsTer46, p.Phe28SerfsTer59, and exon 5-6 deletion [p.(amino acid)].

**Figure 2:**
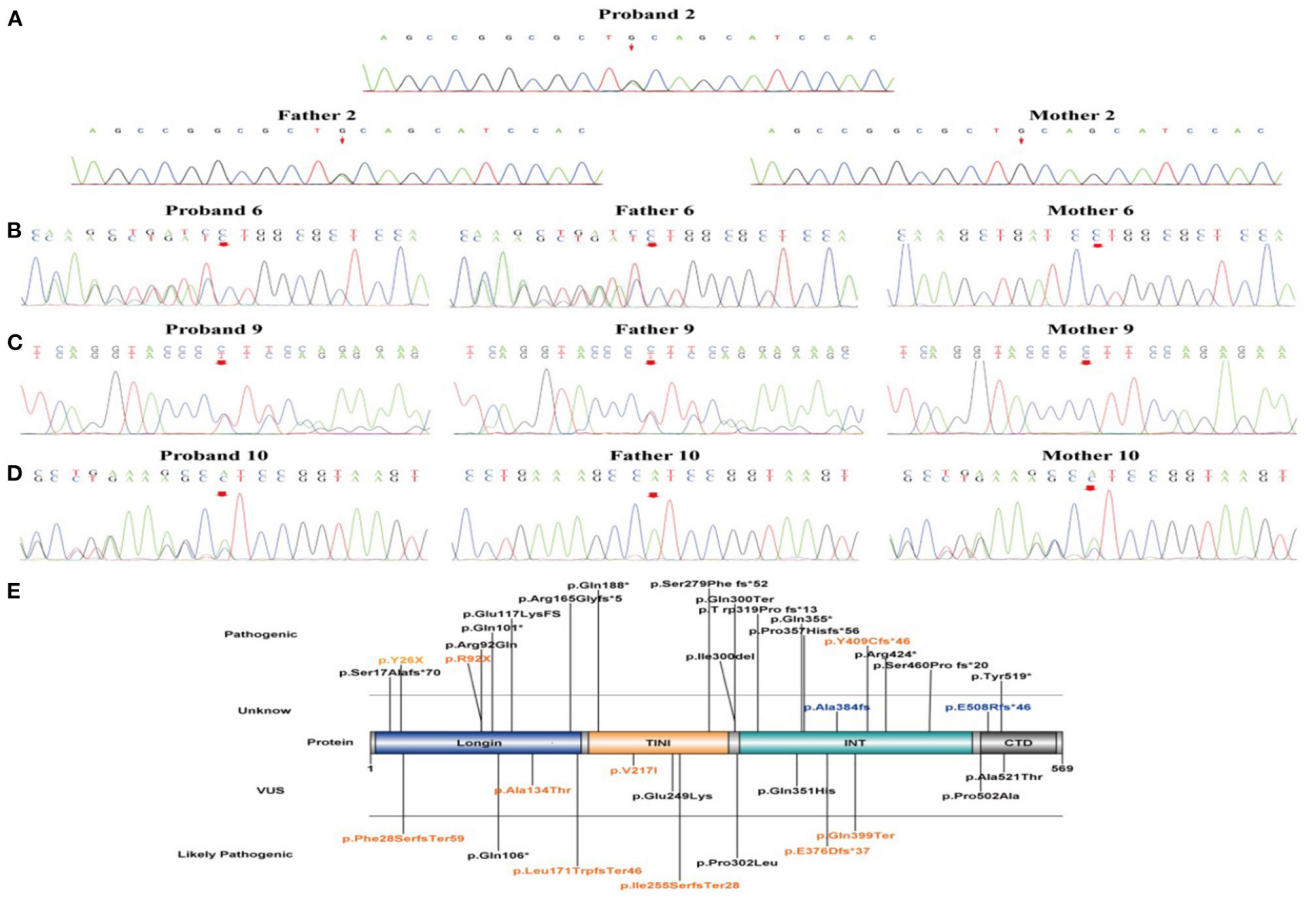
The validation of *NPRL3* variants by Sanger sequencing and schematic diagram of NPRL3 protein. **(A–D)** Sanger sequencing results of the proband 2, 6, 9, and 10 and their parents, respectively. **(E)** Schematic diagram of the variant sites in NPRL3 proteins from our results and previous literature identified. The pathogenic variants identified in this study are illustrated and highlighted in yellow.

Sanger sequencing confirmed that only one case was *de novo* pathogenic variants (case 7, c.1226-1227delAT, and p.Y409Cfs^*^46), and the remaining ten cases were inherited, including seven with paternal inheritance (cases 1, 2, 5, and 8–11) and three with maternal inheritance (cases 3, 4, and 6) (Figures 2A–D). According to the ACMG guidelines (15), three mutations were resolved into pathogenic (cases 1, 3, and 7), five mutations were resolved into probably pathogenic (cases 2, 6, 8, 9, 10, and 11), and two mutations were resolved into VUS (cases 4 and 5). As for two mutations of VUS, the gene variants of cases 4 and 5 were missense variants. In case 4, we found that its gnomAD allele frequencies were lower than the minimum allele frequency (0.0056% < 0.03%), indicating within the pathogenic range threshold. In addition, its mutation was predicted pathogenic by M-CAP. In case 5, its variant site could not be found in normal populations from the ESP database, the 1000 Genome Project, the Genome Aggregation Database, and the Exome Aggregation Consortium, suggesting that the mutation in this site might generate possible pathogenicity. Of the ten variants, two variant sites were located on exon 2, one on exon 4, two on exon 6, two on exon 8, one on exon 11, and two on exon 12. The location of these 10 variant sites on the protein structure schematic is shown in Figure 2E.

### The conservation assessment and protein tertiary structure of *NPRL3* mutation

**Figure 3:**
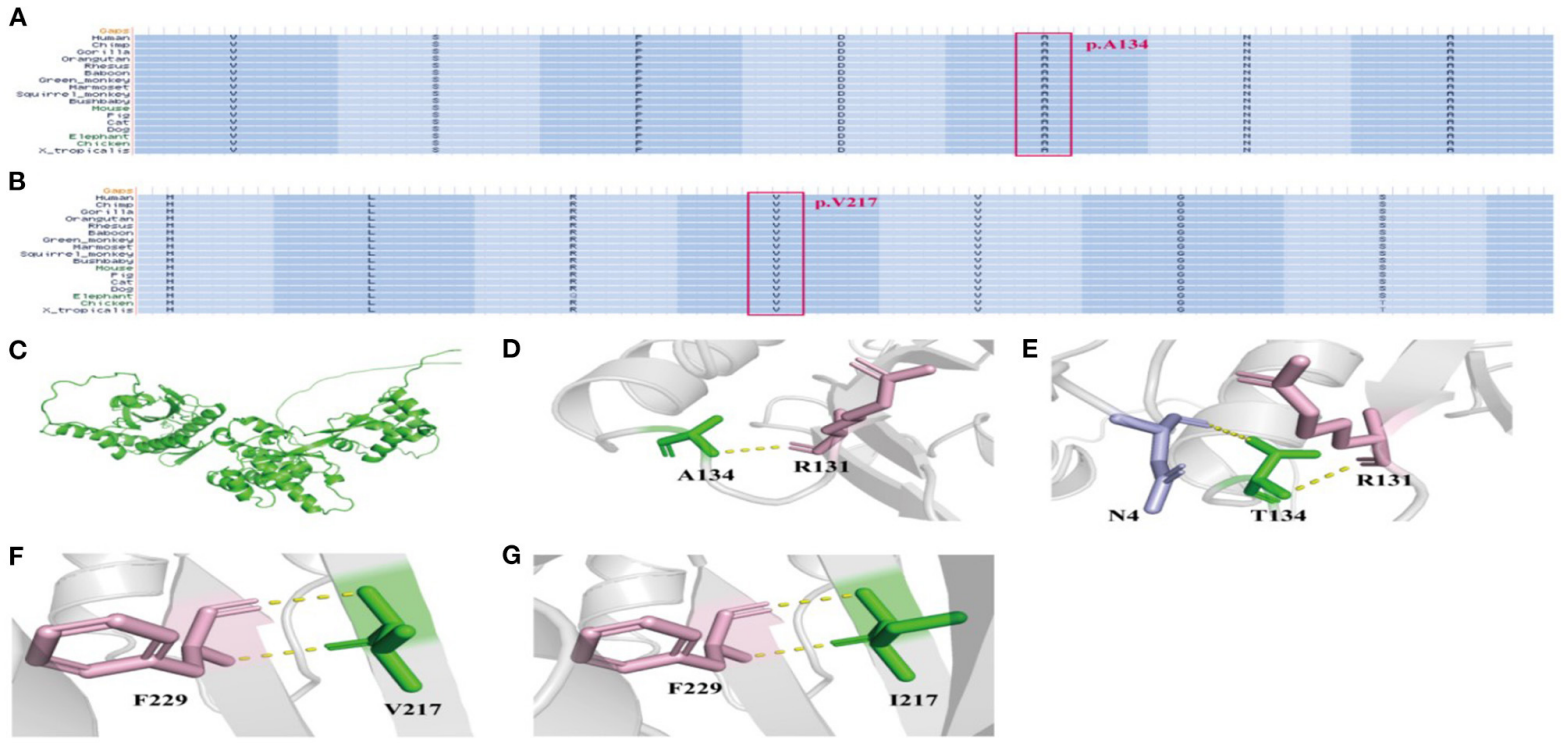
The conservation assessment of *NPRL3* mutation sites and the NPRL3 protein tertiary structure. **(A)** The conservation assessment of c.400G>A (p.Ala134Thr). **(B)** The conservation assessment of c.649G>A (p.Val217Ile). **(C)** The 3D structure of wild type and mutant type of NPRL3 protein structure. **(D, E)** The NPRL3 protein tertiary structure of c.400G>A mutation. **(F, G)** The NPRL3 protein tertiary structure c.649G>A mutations.

In this study, the conservation assessment of the two missense mutation sites (cases 4 and 5) was constructed. We found that p.Ala134 and p.Val217 are highly conserved in different species (Figures 3A, B), suggesting that mutations in these two sites may affect the function of the protein.

We also constructed protein tertiary structure models of NPRL3 (Figure 3C), which could show changes in the spatial structure of the NPRL3 protein after the gene pathogenic variant. In case 4 (c.400G>A and p.Ala134Thr), Ala at position 134 was replaced by Thr, resulting in the formation of an extra hydrogen bond between Thr and N4 in NPRL3 protein (Figures 3D, E). In case 5 (c.649G>A and p.Val217Ile), the amino acid of Val at position 217 was replaced by Ile, and the complex structure of the NPRL3 protein did not change (Figures 3F, G).

### Literature review of previously reported patients

Up to June 2022, 77 patients with *NPRL3-*related epilepsy had been reported previously (Supplementary Table). The age at the onset ranged widely from within hours of birth to 51 years. Among the patients with available information, 78.3% had a family history of epilepsy (54/69). About two-thirds of the patients were diagnosed with focal epilepsy (48/72), including 12 of SHE, 10 of FLE, 7 of TLE, and 19 of unclassified focal epilepsy (UFE). Among the 14 patients who described the abnormal findings of MRI, 9 revealed FCD, of which 8 were with FCD II a. Electroencephalogram (EEG) showed that the initial discharge site of 46.3% of patients was from the frontal lobe (19/41). In addition, 63.4% of the patients had drug-resistant epilepsy (26/41). Among the 11 patients receiving epilepsy surgery, 9 achieved a seizure-free status. Among these mutations, c.275G>A, c.745G>A, and c.1270C>T were more common than the *NPRL3* gene mutations.

## Discussion

The pathogenic variants in genes encoding upstream regulators of the mechanistic target of mTORC1 cause epilepsies and neurodevelopmental disorders (16). NPRL3 is an important component of the GATOR1 complex, and its mutations can promote the activity of the mTOR signaling pathway, thereby causing epilepsy (8, 17, 18). In this study, we reported 11 cases with *NPRL3* gene mutations who presented with different kinds of childhood epilepsy, 8 of whom were new reports, which expanded the clinical and genetic spectrum of *NPRL3*-related epilepsy. SHE and FLE were the most common clinical presentations, and ISs are a new phenotype of *NPRL3*-related epilepsy. The gene mutations or deletion of the 11 patients resulted in haploinsufficiency of the *NPRL3* gene, suggesting a potential genotype-phenotype correlation.

Up to June 2022, a total of 77 patients with *NPRL3*-related epilepsy had been reported in PubMed. Combined with our study, a total of 88 patients with *NPRL3*-related epilepsy were found, of which 57 were boys and 30 were girls, respectively, while the gender of one case was unknown. Among 76 cases with the age of onset, *NPRL3*-related epilepsy could occur from infancy to adulthood (8), suggesting more attention should be given to *NPRL3* gene variations of epilepsy across all ages. The main type of epilepsy due to *NPRL3* gene mutations was focal epilepsy, although epilepsy with tonic–clonic seizures (two cases) and neonatal seizures (one case) was also observed. The most common epileptic phenotype was SHE, which was observed in 17 cases, followed by FLE (12 cases) and TLE (7 cases). A total of 19 cases of unclassified focal epilepsy (UFE) and 21 cases of unclassified epilepsy (UE) were also included. One case presented febrile seizures (FS) in a prior study (19). Indeed, we found two cases of IS as a new phenotype of *NPRL3*-related epilepsy in our study, which complement the clinical phenotype of *NPRL3*-related epilepsy. EEG results showed that the initial discharge site was changeable (18), half from the frontal lobe, which was consistent with the clinical presentations that SHE and FLE were the most phenotype in *NPRL3*-related epilepsy. Previous studies showed that the phenotype of epilepsy was not directly correlated with the affected components in the GATOR l complex, which may be attributed to the co-performing function of the three protein composition complexes (20). Specifically, the clinical phenotypic variability observed in NPRL3 individuals was associated with the link between NPRL3 and mTOR pathway hyperactivation, abnormal neuronal morphology, altered metabolic control of mTOR, disorganized cerebral cortical lamination, and enhanced seizure susceptibility (21).

It was worth noting that most *NPRL3*-related epilepsy exhibited no intracranial structural damage (70%), suggesting that the epileptogenic mechanisms of the *NPRL3* gene may be associated with a relatively wide range of neurofunctional abnormalities and the formation of the epileptogenic network (8). Among the remaining patients with neuroimaging abnormalities, FCD was the common presentation of *NPRL3*-related epilepsy (62.5%), which was consistent with the previous studies (6). However, for those with negative brain MRI results, small FCD may be overlooked. Thus, a PET scan is needed in the future to reveal the subtle metabolic or biochemical function changes in the brain of patients with NPRL3-related epilepsy. The generation mechanism may be that the functional loss of the *NPRL3* gene may cause the abnormal development of filamentous pseudo and dendrites, resulting in the abnormal localization of neurons, and finally causing the structural abnormality of the cerebral cortex in FCD (12). A total of 12 children with definite epileptogenic foci were surgically treated, and 9 were seizure-free post-surgery; thus, surgery represents a highly effective treatment in 75% of the patients with *NPRL3*-related epilepsy. Moreover, the post-operative histological examination revealed eight cases with FCD, of which six were treated effectively after surgery, the same as the above effective rate of surgical treatment. Therefore, these pieces of evidence indicated that epilepsy surgery might be a suitable option for patients of *NPRL3*-related epilepsy with neuroimaging abnormalities, especially with FCD. Meanwhile, a comprehensive presurgical assessment for the identification of neuroimaging abnormalities was required for patients with unclear neuroimaging information.

Our results also showed that 60.8% of patients with *NPRL3*-related epilepsy had drug-resistant epilepsy, consistent with the previous studies (6, 22), which may be due to the high occurrence of FCD among patients with neuroimaging abnormalities (6). Among the 15 patients with monotherapy-controlled seizures, 11 were controlled by sodium channel blockers, including 4 with CBZ, 4 with OXC, and 3 with LTG. Thus, the above results suggested that sodium channel blockers may be the preferred treatment for *NPRL3*-related epilepsy. Additionally, two cases had ongoing seizures after surgery or ASMs but were treated effectively by receiving KD, suggesting that KD may be a potential option for patients of *NPRL3*-related epilepsy unresponsive to surgery or ASMs. However, given our limited cases, future studies need more patients with *NPRL3*-related epilepsy to confirm this. Moreover, the underlying molecular mechanism suggests that mTOR inhibitors, such as rapamycin, may be promising drugs for *NPRL3*-related epilepsy, due to the abnormal activation of the mTOR signaling pathway caused by *NPRL3* gene variation (8, 23, 24). However, two cases in the previous reports were not controlled by sirolimus (17, 25), one of which was treated effectively in the first 3 months, but stopped due to the intermittent diaper rashes, eczema, or respiratory infections, allowing time for the patient to prepare for surgery (17). Although the therapeutic efficacy of the mTOR suppressant drug was uncertain, it could still be considered as a bridging therapy until surgery can be performed in patients either non-responsive or only partially responsive to other antiepileptic drugs (25). In the future, further explorations are still needed to ascertain the stage of rapamycin treatment.

Among the total of 88 cases reported, which included both our study and the previous studies, we found that a majority of patients had *NPRL3* variants inherited from an unaffected parent, suggesting that autosomal dominant inheritance with incomplete penetrance was a prominent feature of *NPRL3*-related epilepsy. Nevertheless, previous studies showed that family members with the same genotype presented different phenotypes of epilepsy (14). The possible explanation for this feature was that the epigenetic regulation and the second hit during brain development may greatly affect the variability of the phenotypes (12). We also found that the distribution of mutation types in the *NPRL3* gene was mainly LOF variants (nonsense mutations, frameshift mutations, and exons deletion) that account for 75% (12, 14). Because the pathogenesis of *NPRL3* gene variation was haploinsufficiency, patients with LOF variants have a severe clinical phenotype of epilepsy (14). All six patients with deletion of the *NPRL3* gene had drug-resistant epilepsy, suggesting that the phenotype of the deletion variant may be the most severe variant of LOF in *NPRL3* gene variations. Among these variants, c.275G>A, c.745G>A, and c.1270C>T were more common than others. The pathogenic mechanism of these common variants was the proposed mechanism of nonsense mutation-mediated mRNA decay and consequently increased the expression of downstream molecular Phospho-p70 S6 kinase (P-s6k), resulting in increased mTOR pathway activity, ultimately causing the occurrence of epilepsy (11–13).

## Conclusion

In this study, we reported 11 Chinese patients with *NPRL3-*related epilepsy, 8 of whom had not previously been reported, and found ISs as a new phenotype of *NPRL3*-related epilepsy, expanding the clinical and molecular genetic spectrum of *NPRL3*-related epilepsy. Based on the literature review, SHE and FLE are the most common clinical presentations, always with a high rate of drug resistance. Sodium channel blockers may be the preferred treatment for *NPRL3*-related epilepsy. Patients with FCD are considered good surgical candidates who may achieve a seizure-free status post-surgery. KD and rapamycin may be potential treatments for patients unresponsive to ASMs or surgery. Among the mutations, c.275G>A, c.745G>A, and c.1270C>T were more common, which may be helpful in molecular genetic analysis.

## Data availability statement

The datasets presented in this study can be found in online repositories. The name of the repository and accession number can be found below: GenBank (https://www.ncbi.nlm.nih.gov/genbank/), accession numbers OQ442812-442820.

## Ethics statement

All procedures of this study were approved by the Ethics Committee of Beijing Children's Hospital, Capital Medical University (2019-k-262). Written informed consent to participate in this study was provided by the participants' legal guardian/next of kin. Written informed consent was obtained from the individual(s), and minor(s)' legal guardian/next of kin, for the publication of any potentially identifiable images or data included in this article.

## Author contributions

HZ wrote the first draft. JD, XW, and CC collected the data and completed the data analysis. SC and LD contributed to the conception of the work and revised it critically for important intellectual content. FF revised this manuscript and approved it for submission. All authors contributed to the article and approved the submitted version.
