# Supplementary material for: Clinical phenotypic and genotypic characterization of NPRL3-related epilepsy
Source: Front Neurol. 2023 Mar 2;14:1113747. doi: 10.3389/fneur.2023.1113747 (PMC10018541; doi:10.3389/fneur.2023.1113747)
Supplement: Supplementary file 1 [file Table_1.docx]

**Supplementary Table |** The genotype and clinical characteristics of patients with *NPRL3*-related epilepsy from previous studies.

| Reference | Sex | Case  No. | cDNA variant | Protein alteration | Variant Class | ACMG classification | Epilepsy syndrome | Epilepsy surgery | SUDEP in the family | Inheritance | Seizure outcome | MRI | EEG | ASMs | Drug-resistant | Age at onset | Age studied | Family history of epilepsy |
| --- | --- | --- | --- | --- | --- | --- | --- | --- | --- | --- | --- | --- | --- | --- | --- | --- | --- | --- |
| Baldassari S, et al. | M | 1 | c.1557C>G | p.Tyr519* | Nonsense | P | SHE | No | No | N/A | Seizure free | Normal | Left frontal epileptic discharges | CBZ, VPA, CLB | No | 4y 6m | 5y 6m | No |
|  | M | 2 | c.1270C>T | p.Arg424* | Nonsense | P | SHE | No | No | Inherited | Drug resistant for 4y | Normal | Right frontal epileptic discharges | VPA, LEV | Yes | 10y | 19y | Yes |
|  | F | 3 | c.562C>T | p.Gln188* | Nonsense | P | SHE | No | Yes | Inherited | Seizure free | Normal | Right frontal epileptic discharges | CBZ | No | 16y | 38y | Yes |
|  | F | 4 | c.493delC | p.Arg165Glyfs*5 | Frameshift | P | UFE | No | N/A | Inherited | Seizure free | Normal | N/A | VPA, LTG | No | 1y | 10y | Yes |
|  | F | 5 | c.301C>T | p.Gln101* | Nonsense | P | UFE | No | No | Inherited | Drug resistant for 10y | Normal | Left centro-parietal region | LEV, TPM, VPA, PB, LTG, ZSM | Yes | 1y | 19y | Yes |
|  | F | 6 | Deletion (exons 5-10) | p.(?) | Exon deletion | P | FLE | No | No | Inherited | Seizure free | Normal | Interictal: initial EEGs with right and later EEGs with left fronto-central spikes. | VPA, CBZ, LTG | Yes | 6y | 12y | Yes |
|  | F | 7 | Deletion (exons 1-7) | p.(?) | Exon deletion | P | UFE | No | No | Inherited | Seizure free | Normal | Interictal: left central epileptiformabnormalities. Ictal: left central electroclinical sz. | PHT, OXC, LCM, BZD, LEV, KD | Yes | 13m | 22m | Yes |
| Iffland PH, et al. | N/A | 8 | c.349delG | p.Glu117LysFS | Frameshift | P | N/A | N/A | N/A | N/A | N/A | Normal | Nineteen EEGs showed both focal and generalized abnormalities while two individuals had normal EEGs | N/A | N/A | N/A | N/A | N/A |
| Li Y, et al. | F | 9 | c.316C>T | p.Gln106* | Nonsense | LP | Unclassified | N/A | N/A | Inherited | Seizure free | Brain atrophy | Normal | PB | No | 8y | 74y | Yes |
|  | M | 10 | c.316C>T | p.Gln106* | Nonsense | LP | TLE | N/A | N/A | Inherited | 10 seizures per year | Normal | Sharp and wave complex predominantly in right temporal lobe | CZP, PHT, VPA | Yes | 16y | 42y | Yes |
|  | M | 11 | c.316C>T | p.Gln106* | Nonsense | LP | Unclassified | N/A | N/A | Inherited | 10 epileptic auras per year | Normal | Normal | PB | N/A | 9y | 41y | Yes |
|  | M | 12 | c.316C>T | p.Gln106* | Nonsense | LP | Unclassified | N/A | N/A | Inherited | 1-2 seizures per year | Normal | Normal | PB, CBZ | Yes | 20y | 37y | Yes |
|  | M | 13 | c.316C>T | p.Gln106* | Nonsense | LP | FLE | N/A | N/A | Inherited | Seizure free | Normal | Sharp waves in the right frontal lobe; normal later | LTG | No | 19y | 28y | Yes |
| Abumurad S, et al. | M | 14 | c.1270C > T | p.Arg424* | Nonsense | P | Unclassified | Yes（LITT） | N/A | Inherited | Seizure free | Normal | Heterogeneous interictal epileptiform discharges (IEDs) in the left temporal, frontotemporal and orbitofrontal regions | LTG, LEV, VPA, OXC, CLB and ZNS | Yes | 24y | 27y | Yes |
| Canavati C, et al. | M | 15 | 38 kb 16p13.3g.(114012‐151819) | p.(?) | Copy number loss | P | SHE | No | N/A | N/A | Ongoing seizures | N/A | N/A | CBZ, VPA | Yes | 10y | 60y | Yes |
|  | F | 16 | 38 kb 16p13.3g.(114012‐151819) | p.(?) | Copy number loss | P | UFE | No | N/A | N/A | Ongoing seizures | N/A | N/A | CBZ | N/A | 30y | 52y | Yes |
|  | F | 17 | 38 kb 16p13.3g.(114012‐151819) | p.(?) | Copy number loss | P | Unclassified | No | N/A | Inherited | Seizure free | N/A | No IED, intermittent left posterior and generalized slowing | CBZ | No | 9y | 12y | Yes |
|  | M | 18 | 38 kb 16p13.3g.(114012‐151819) | p.(?) | Copy number loss | P | Unclassified | No | N/A | Inherited | N/A | N/A | No IED, intermittent left frontotemporal slowing | LTG | N/A | 32y | 34y | Yes |
|  | F | 19 | 38 kb 16p13.3g.(114012‐151819) | p.(?) | Copy number loss | P | UFE | No | N/A | Inherited | Ongoing seizures | Normal | No IED, intermittent bilateral posterior and right frontal slowing | CBZ | N/A | 11y | 31y | Yes |
|  | F | 20 | 38 kb 16p13.3g.(114012‐151819) | p.(?) | Copy number loss | P | FLE | No | N/A | Inherited | Seizure free | N/A | Right frontal IED, inter- mittent bilateral parietal slowing | CBZ | No | 2y | 20y | Yes |
|  | M | 21 | 38 kb 16p13.3g.(114012‐151819) | p.(?) | Copy number loss | P | UFE | No | N/A | Inherited | Ongoing seizures | N/A | N/A | CBZ | N/A | 17y | 18y | Yes |
|  | F | 22 | 38 kb 16p13.3g.(114012‐151819) | p.(?) | Copy number loss | P | UFE | Yes | N/A | Inherited | Ongoing seizures | N/A | N/A | VPA,LTG | Yes | 3m | 13y | Yes |
|  | M | 23 | 38 kb 16p13.3g.(114012‐151819) | p.(?) | Copy number loss | P | Unclassified | No | N/A | Inherited | Seizure free | Normal | Normal | VPA | No | 1y | 4y | Yes |
|  | F | 24 | 38 kb 16p13.3g.(114012‐151819) | p.(?) | Copy number loss | P | UFE | No | N/A | Inherited | Ongoing seizures | Right side hemime-galencep-haly with dysplastic cortex | Right hemisphere IED, generalized and right hemisphere slowing | VPA, CBZ | Yes | 0m | 6m | Yes |
|  | M | 25 | c.1063C>T | p.Gln355* | Nonsense | P | Unclassified | N/A | N/A | Inherited | Ongoing seizures | N/A | No | No | N/A | 14y | 16y | Yes |
|  | M | 26 | c.1063C>T | p.Gln355* | Nonsense | P | UFE | N/A | N/A | Inherited | Seizure free | N/A | Diffuse slowing | No | No | 9y | 12y | Yes |
|  | M | 27 | c.1063C>T | p.Gln355* | Nonsense | P | Unclassified | N/A | N/A | Inherited | Seizure free | N/A | Left frontotemporal slowing | No | N/A | 7y | 41y | Yes |
|  | M | 28 | c.1063C>T | p.Gln355* | Nonsense | P | UFE | N/A | N/A | Inherited | Ongoing seizures | N/A | Fast activity in the left frontotemporal and central region | CBZ, OXC, VPA, PHT | Yes | 5y | 21y | Yes |
|  | M | 29 | c.1063C>T | p.Gln355* | Nonsense | P | UFE | N/A | N/A | Inherited | Ongoing seizures | N/A | Diffuse slowing more prominent on the left | VPA, LTG, OXC, PB | Yes | 3y | 9y | Yes |
| Chandrasekar I, et al. | F | 30 | Deletion/ (exon 5) | p.(?) | Exon deletion | LP | N/A | Yes | N/A | Inherited | Seizure free | Hemimegalencephaly | Seizures in the left hemisphere along the midline, occasionally originating from right hemisphere | PB, LEV, PHT, pyridoxine, TPM, midazolam infusion, CLB, sirolimus | Yes | 1d | N/A | No |
| Vawter-Lee M, et al. | M | 31 | Heterozygous deletionwithin 16p13.3 of 552 kb | p.(?) | Deletion | P | Unclassified | Yes | N/A | Inherited | Seizure free | Large left hemisphere cortical malformation | burst-suppression pattern with chaotic background | PB, OXC, LEV, PHT, pyridoxine, ACTH, VGB, sirolimus | Yes | within hours of birth | N/A | Yes |
| Ricos MG, et al. | M  (4) | 32-35 | c.835_836insT | p.Ser279Phe fs*52 | Frameshift | P | 2 SHE, 1 Neonatal seizures, 1 FS | N/A | N/A | Inherited | N/A | N/A | N/A | N/A | N/A | N/A | N/A | Yes |
|  | M (3) | 36-38 | c.1375_1376insAC | p.Ser460Pro fs*20 | Frameshift | P | 2 TLE, 1 Nocturnal TCS | N/A | N/A | N/A | N/A | N/A | N/A | N/A | N/A | N/A | N/A | Yes |
|  | F | 39 | c.745G>A | p.Glu249Lys | Missense | P | UFE | N/A | N/A | De novo | N/A | N/A | N/A | N/A | N/A | N/A | N/A | Yes |
|  | M | 40 | c.275G>A | p.Arg92Gln | Missense | P | FLE | N/A | N/A | N/A | N/A | N/A | N/A | N/A | N/A | N/A | N/A | Yes |
|  | F | 41 | c.954_955insCCCA | p.T rp319Pro fs*13 | Frameshift | P | TLE | N/A | N/A | N/A | N/A | N/A | N/A | N/A | N/A | N/A | N/A | Yes |
| Sim JC, et al. | M | 42 | c.1375_1376dupAC | p.S460Pfs*20 | Frameshift | P | N/A | Yes | N/A | Inherited | Ongoing seizures | Right posterior quadrantic dysplasia（FCDIIa） | Suppression burst, more prominent on the right | OXC | N/A | 1d | N/A | Yes |
|  | M | 43 | c.1375_1376dupAC | p.S460Pfs*20 | Frameshift | P | Unclassified | No | N/A | Inherited | Seizure free | Normal | left frontal sharp waves | OXC | No | 2y | N/A | Yes |
|  | F | 44 | c.1375_1376dupAC | p.S460Pfs*20 | Frameshift | P | SHE | Yes | N/A | Inherited | Seizure free | bottomof-sulcus dysplasia in the right cingulate sulcus（FCDIIa） | ictal rhythms and postictal slowing over the right frontal region | OXC | N/A | 7y | N/A | Yes |
|  | M | 45 | c.1375_1376dupAC | p.S460Pfs*20 | Frameshift | P | SHE | No | N/A | Inherited | Seizure free | Normal | continuous slowing, focal interictal epileptiform discharges, and seizures of right frontal origin | OXC | No | 4y | N/A | Yes |
|  | M | 46 | c.1352-4delA-CAGinsTGACCCATCC | p.(?) | possible Splice-region | N/A | UFE | Yes | N/A | De novo | Seizure free | Extensive left frontal operculum and insula dysplasia（FCDIIa） | Left central interictal and ictal abnormalities | N/A | Yes | 4m | N/A | No |
|  | F | 47 | c.275G>A | p.R92Q | Missense | VUS | N/A | Yes | N/A | N/A | Seizure free | Diffuse dysplasia in the left central region（FCDIIa） | Normal | CBZ | Yes | 15m | N/A | Yes |
| Korenke GC, et al. | F | 48 | c.1522delG | p.E508Rfs*46 | Frameshift | N/A | SHE | N/A | N/A | Inherited | Seizure free periods: 3y-13y, 15–20y, 29–36 y | Normal | Unknown | LTG | N/A | 2y | 36y | Yes |
|  | M | 49 | c.1522delG | p.E508Rfs*46 | Frameshift | N/A | SHE | N/A | N/A | Inherited | Ongoing seizures | Normal | Precentral, temporal sharp waves (4 y) | TPM | N/A | 12y | 18y | Yes |
|  | M | 50 | c.1522delG | p.E508Rfs*46 | Frameshift | N/A | SHE | N/A | N/A | Inherited | Ongoing seizures | Normal (12y and 25y), right frontal lobe:small subcortical signal enhancement (31y) | Right temporal sharp-waves (4y), right frontal sharp slow waves (7y) frontocentral spikes (20y) | LTG, VPA, PER | Yes | 4y | 23y | Yes |
| Dunn PJ, et al. | M | 51 | c.1504C > G | p.Pro502Ala | Missense | VUS | Unclassified | N/A | N/A | N/A | N/A | N/A | N/A | N/A | Yes | N/A | 15y 4m | N/A |
| Weckhuysen S, et al. | M | 52 | c.1070delC | p.Pro357Hisfs*56 | Frameshift | P | FLE | N/A | No | Inherited | N/A | Right frontoparietal FCD  （FCD IIb） | Bilateral frontal spikes. Fast left frontal ictal activity | VPA, CBZ | N/A | 2y | 5y | Yes |
|  | M | 53 | c.1070delC | p.Pro357Hisfs*56 | Frameshift | P | Epilepsy with tonic– clonic seizures | N/A | N/A | Inherited | Seizure free | N/A | Normal | VPA | No | 4.5y | 6y | Yes |
|  | M | 54 | c.1070delC | p.Pro357Hisfs*56 | Frameshift | P | FLE | N/A | N/A | Inherited | N/A | Normal | Right fronto(parieto)temporal discharges | VPA, LEV, CBZ | N/A | 3.5y | N/A | Yes |
|  | F | 55 | c.1070delC | p.Pro357Hisfs*56 | Frameshift | P | Epilepsy with tonic– clonic seizures | N/A | N/A | Inherited | Ongoing seizures | N/A | Left temporal epileptic activity | OXC | N/A | 14y | N/A | Yes |
|  | M | 56 | c.1070delC | p.Pro357Hisfs*56 | Frameshift | P | TLE | N/A | N/A | Inherited | Seizure free | N/A | N/A | CBZ | No | 51y | N/A | Yes |
|  | F | 57 | c.1270C>T | p.Arg424* | Nonsense | P | FLE | N/A | Yes | Inherited | Ongoing seizures | Normal | Normal | N/A | Yes | 8y | 43y | Yes |
|  | F | 58 | c.1270C>T | p.Arg424* | Nonsense | P | UFE | N/A | SUDEP | Inherited | N/A | N/A | N/A | N/A | Yes | 10m | N/A | Yes |
|  | M | 59 | c.1270C>T | p.Arg424* | Nonsense | P | Unclassified | Yes | Yes | Inherited | Seizure free | left frontal FCD(FCD IIa) | Left frontocentral spikes | N/A | N/A | 2m | N/A | Yes |
|  | F | 60 | c.1270C>T | p.Arg424* | Nonsense | P | Unclassified | N/A | Yes | Inherited | N/A | N/A | Diffuse atypical and irregular spike wave complexes | N/A | N/A | N/A | N/A | Yes |
|  | M | 61 | c.1270C>T | p.Arg424* | Nonsense | P | Unclassified | N/A | Yes | Inherited | N/A | N/A | Clear generalized spike wave discharges | N/A | N/A | N/A | N/A | Yes |
|  | M | 62 | c.1270C>T | p.Arg424* | Nonsense | P | Unclassified | N/A | Yes | Inherited | Seizure free | N/A | Diffuse spike wave activity | PB | No | 5y | 31y | Yes |
|  | M | 63 | c.1270C>T | p.Arg424* | Nonsense | P | UFE | N/A | Yes | Inherited | N/A | N/A | N/A | N/A | N/A | N/A | N/A | Yes |
|  | M | 64 | c.1270C>T | p.Arg424* | Nonsense | P | Unclassified | N/A | Yes | Inherited | N/A | N/A | N/A | N/A | N/A | 12y | N/A | Yes |
|  | F | 65 | c.1270C>T | p.Arg424* | Nonsense | P | UFE | N/A | Yes | Inherited | N/A | N/A | N/A | N/A | N/A | N/A | N/A | Yes |
|  | M | 66 | c.1270C>T | p.Arg424* | Nonsense | P | Unclassified | N/A | Yes | Inherited | N/A | N/A | N/A | N/A | N/A | N/A | N/A | Yes |
| Bennett MF, et al. | M | 67 | c.48delG | p.Ser17Alafs*70 | Frameshift | P | SHE | Yes | N/A | Inherited | Ongoing seizures | Abnormal gray matter in the right posteromesial frontal region（FCD IIA） | Repetitive spike-and-wave activity in the right frontocentral region, followed by attenuation and rapid spread to bilateral frontocentral regions with low-amplitude fast activity evolving to rhythmic theta activity. | N/A | Yes | 2y | 10y | N/A |
|  | M | 68 | c.48delG | p.Ser17Alafs*70 | Frameshift | P | FLE | Yes | N/A | Inherited | Seizure free | Left anteromesial frontal cortical abnormality（FCD IIA） | Left frontal focus | N/A | Yes | 6w | 6y | N/A |
| Blümcke I, et al. | F | 69 | c.1149dupC | p.Ala384fs | Frameshift | N/A | N/A | Yes | N/A | N/A | Seizure free | Left frontal（PMG） | N/A | N/A | N/A | 6y | N/A | N/A |
| Krenn M, et al. | F | 70 | c.905C>T | p.Pro302Leu | Missense | LP | FLE | N/A | N/A | N/A | Drug-resistant | Normal | Ictal: bilateral frontal Interictal: no changes | PRG, LTG, CBZ, ZNS | Yes | 1y | N/A | Yes |
|  | M | 71 | 380 kb microdeletion, 16p13.3 | N/A | Deletion | P | UFE | N/A | N/A | N/A | Drug-resistant | Normal | Ictal: right hemispheric Interictal: right frontotemporal spikes | CBZ, LEV, TPM | Yes | 10y | N/A | No |
|  | M | 72 | c.898C>T | p.Gln300Ter | Nonsense | P | FLE | N/A | N/A | N/A | Drug-resistant | Normal | Ictal: not localisable Interictal: no changes | PHT , LEV , LCM, RUF, ZNS, CBZ, VPA, LTG, PER, VGB, PRM, DBS implant | Yes | 3y | N/A | No |
|  | F | 73 | c.1561G>A | p.Ala521Thr | Nonsense | VUS | TLE | N/A | N/A | N/A | N/A | Normal | N/A | N/A | N/A | 6y | N/A | N/A |
|  | M | 74 | c.745G>A | p.Glu249Lys | Missense | VUS | TLE | N/A | N/A | N/A | N/A | Normal | N/A | N/A | N/A | 14y | N/A | N/A |
|  | M | 75 | c.1053G>C | p.Gln351His | Missense | VUS | Unclassified | N/A | N/A | N/A | N/A | Normal | N/A | N/A | N/A | 23y | N/A | N/A |
|  | F | 76 | c.898-900del | p.Ile300del | In-frame indel | P | UFE | Yes | N/A | Inherited | Seizure free | Left frontal lobe(FCD IIa) | Ictal onset in the left central area | LEV, VGB | Yes | 0m | N/A | Yes |
|  | F | 77 | c.898-900del | p.Ile300del | In-frame indel | P | UFE | No | N/A | Inherited | Seizure free | Normal | N/A | OXC | No | 13y | N/A | Yes |

**Abbreviations:** BZD, benzodiazepine; CBZ, carbamazepine; CLB, clobazam; CZP, clonazepam; DBS, deep brain stimulation; EEG, electroencephalogram; M, Male; F, female; FCD, focal cortical dysplasia; FE, focal epilepsy; FFEVF, familial focal epilepsy with variable foci; FLE, frontal lobe epilepsy; FS, febrile seizure; IED, interictal epileptiform discharge; IS, infantile spasms; KD, ketogenic diet; LCM, lacosamide; LEV, Levetiracetam; LITT, Laser interstitial thermal therapy; LP, likely pathogenic; LTG, lamotrigin; N/A, not applicable; OXC, oxcarbazepine; P, pathogenic; PB, phenobarbital; PER, perampanel; PHT, phenytoin; PMG, polymicrogyria; PRG, pregabalin; PRM, primidone; RUF, pufinamide; SHE, sleep-related hypermotor epilepsy; SUDEP, sudden unexpected death in epilepsy; TLE, temporal lobe epilepsy; TPM, topiramate; UFE, unclassified focal epilepsy; VGB, vigabatrin; VPA, valproic acid; VUS, variant of uncertain significance; ZNS, zonisamide; TCS, tonic clonic seizures.
